# Optimization of a molten iron scheduling problem with uncertain processing time using variable neighborhood search algorithm

**DOI:** 10.1038/s41598-022-10891-9

**Published:** 2022-05-04

**Authors:** Linyu Liu, Zhiqi Chang, Shiji Song

**Affiliations:** 1 Department of Automation, Tsinghua University, Beijing, 100084 China; 2 Department of Artificial Intelligence, Cainiao Network, Hangzhou, 311100 China

**Keywords:** Applied mathematics, Computational science, Electrical and electronic engineering

## Abstract

Punctuality of the steel-making scheduling is important to save steel production costs, but the processing time of the pretreatment process, which connects the iron- and steel-making stages, is usually uncertain. This paper presents a distributionally robust iron-steel allocation (DRISA) model to obtain a robust scheduling plan, where the distribution of the pretreatment time vector is assumed to belong to an ambiguity set which contains all the distributions with given first and second moments. This model aims to minimize the production objective by determining the iron-steel allocation and the completion time of each charge, while the constraints should hold with a certain probability under the worst-case distribution. To solve problems in large-scale efficiently, a variable neighborhood algorithm is developed to obtain a near-optimal solution in a short time. Experiments based on actual production data demonstrate its efficiency. Results also show the robustness of the DRISA model, i.e., the adjustment and delay of the robust schedule derived from the DRISA model are less than the nominal one.

## Introduction

Due to the large energy consumption in the iron- and steel-making process, well-designed scheduling solutions are needed to save energy and increase production efficiency. The classical process of producing steel can be divided into three main stages: (i) iron-making in blast furnaces, (ii) steel-making and continuous-casting (SCC), and (iii) rolling and finishing mills. Since steel needs to be maintained in a liquid state after it is released from the blast furnace and before being continuously cast, scheduling in these processing steps has a significant impact on energy consumption. Thus, the molten iron–steel allocation scheduling, which plays a key role in connecting the iron- and steel-making stages, directly affects the production efficiency and energy consumption.

In the iron-making stage, molten iron is melted from iron ore, limestone and bituminous coal in blast furnaces. Then the molten iron will be poured into *pots* carried on torpedo cars and then be transported to a pretreatment site. The process of pretreatment includes a set of processing steps such as pre-processing, desulphurization and dephosphorization, and post-processing. Which steps will be taken and how long the pretreatment time will be depend on the product specifications, molten iron compositions, and other factors. After that, pots of pretreated molten iron are transported by torpedo cars and poured into converters in the steel-making stage.

The process of production planning for the iron-making and steel-making stages is described as follows. First, according to the customer demands, the steel company should decide how many charges are going to be processed and what the concrete production requirements of each charge should be in the steel-making stage, where *charge* is the basic processing unit in the SCC stage. In the steel-making stage, charges are first processed by one of several converters (e.g., 5 for Baosteel^1^ China). Then, through rough SCC scheduling, the charge sequence on each converter and the processing time of each charge are determined according to the production requirements. Only after the production information of these charges is given does the molten-iron scheduling plan start to be made.

The whole molten-iron scheduling problem is very complex and therefore often decomposed and solved in the following three steps. First, each pot of molten iron should be allocated to a charge in the steel-making stage and it is a one-to-one match. Only after that could steps in the pretreatment of each pot be settled down according to the specific requirement of the needed charge. Second, because the steps (processing routing) that each pot would take in the pretreatment site may be different, and for each processing step there might be several parallel machines, machine assignments and pot processing sequence on each machine are going to be determined in this step. Third, transportation routes of torpedo cars would be established. This paper considers the first step in the molten-iron system, i.e., the molten iron-steel allocation problem with minimizing the total weighted completion time in the converters, and views all the processing steps in the pretreatment site as a whole, because this problem lies on the top layer in iron scheduling systems and directly influence the production efficiency in the steel-making stage.

The length of pretreatment time of a pot depends on which charge it is prepared for, but there are many real-time factors influencing the actual processing time, such as the capacities of machines and cars, components deviation, transportation times, and temperatures. Thus, it is an unavailable value for the dispatcher in advance. Meanwhile, the punctuality of the schedule is significant for the downstream continuous casting production: charges in the same predefined batch have to be continuously cast without any break, or a great fixed cost would be paid for it. Therefore, unexpected disturbances might cause great loss, and solutions for uncertainty are necessary.

Most of the previous work for iron and steel production scheduling focused on the SCC scheduling and hot rolling scheduling, and the literature of molten iron scheduling mainly focused on machine scheduling for the multiple processing steps in the pretreatment and the scheduling of torpedo cars, where the molten iron-steel allocation solution is assumed to be given. For example, Tang et al.^1^ considered the scheduling problem of allocating pots to pretreatment processors. They viewed it as a parallel machine scheduling problem with time windows and designed a branch-and-price algorithm to solve it. Li et al.^2^ considered the scheduling problem of multiple processing steps in the pretreatment. They viewed it as a specific flow-shop scheduling problem and adopted a meta-heuristic algorithm, the artificial bee colony, to solve this complex problem. Ge et al.^3^ utilized an ant colony, which is also a meta-heuristic algorithm, to dispatch torpedo cars^4^. presented a molten iron logistics balance model in iron-steel correspondence scheduling, but what they were concerned about in their model were the weights of molten iron sent from each blast furnace to each steel plant instead of the processing time points. In this paper, a mixed-integer programming model is first presented for this molten iron-steel allocation problem while minimizing the total weighted completion time of charges on converters, which is a direct reflection of maximizing production efficiency. Furthermore, the production uncertainty of processing time in the pretreatment site is considered in this paper.

Usually, solutions for scheduling in an uncertain environment can be classified into two types: proactive and reactive scheduling methods^5^. Proactive scheduling tries to provide a solution to avoid future changes to the initial schedule when disturbances are revealed, so it is suitable to deal with small and frequent fluctuations of the uncertain parameters. Reactive scheduling^6^ dynamically reacts to each unexpected but significant disruption (e.g., machine breakdown) by rescheduling and is more suitable for unusual events. This paper aims to develop a robust scheduling solution to immune small daily disturbances, such that fewer adjustments will be needed in further implementation. To guarantee the feasibility of the solution under uncertainty, this paper employs a *chance constrained model*, i.e., constraints are guaranteed to hold in a certain probability. However, the exact distribution of the uncertain processing time is unknown in practice, but the mean and variance are easy to be obtained from historical data or expert experience. Thus, a moment-based distributionally robust (DR) approach is adopted to deal with it.

The DR method considers that the true distribution of the uncertain parameters belongs to a family of distributions, which are contained in an ambiguity set, and the model aims to optimize the objective function under the *worst-case distribution* in this ambiguity set, instead of the *worst-case scenario* of the uncertain parameter in the uncertainty set in the classic robust optimization approach (see the next section). This paper views the pretreatment times as a random variable vector with known mean and variance. An ambiguity set containing all the distribution with these attributes is established, and a chance-constrained DR model is proposed, where the uncertainty is incorporated through constraints. Then, this chance-constrained DR model is reformulated as a tractable mixed integer programming model. By solving this MIP model, a robust solution containing the allocation result and completion times can be obtained, where the robustness is reflected in the fact that the solution maintains feasible with a certain (given) probability for any distribution in the ambiguity set, and this can be guaranteed by theory.

Although the reformulated MIP model can be solved by off-the-shelve solvers, the solution time for an optimal solution may be unacceptable for a large-scale problem, because it is essentially a special non-identical parallel machine scheduling problem. *Non-identical parallel machine scheduling* refers to the type of problems that the processing times of a job on different machines are different. The ease of solving our special problem lies in the fact that the number of charges to be processed by each machine is given, while the complexity is the processing time in the previous stage depends on the allocation result in the next stage. Computation efficiency for exact solutions to non-identical parallel machine scheduling problems is often low when the problem size grows. Literature on the solution to this is reviewed in the “Related works” section. Therefore, this paper considers obtaining a near-optimal solution by using a meta-heuristic approach, variable neighborhood search (VNS), to take both the computation efficiency and optimality into consideration. Numerical experiments demonstrate the computation efficiency of this algorithm compared with a commercial solver.

The contributions of this paper are summarized as follows.

- This paper considers the molten iron-steel allocation problem and presents a MIP model for it, which can be viewed as a special non-identical parallel machine scheduling problem.
- Pretreatment times of pots are regarded as a random variable vector, whose distribution is assumed to belong to an ambiguity set which contains all the distributions with the same known mean and variance. A chance-constrained DR molten iron-steel allocation model is constructed, in which constraints are guaranteed to hold within a certain probability for any distribution in this ambiguity set. Then, it is reformulated into a tractable MIP form.
- For large-scale instances, a customized VNS algorithm is developed to find a near-optimal solution in a short time. Furthermore, the JVNS with a jump operation is designed and added to the VNS algorithm is provided to avoid premature convergence.
- Experiments on real-world data from a steel company in China demonstrate the robustness of the DR model and the computation efficiency of the meta-heuristic algorithms.

The rest of the paper is organized as follows. In the “Related works” section, literature about machine scheduling algorithms and approaches to deal with uncertainty is reviewed. In the “Problem statement and formulations” section, the DR model and its MIP reformulation are developed. The VNS algorithm and its enhancement are introduced in the “Solution algorithm” section, and the computational experiments and results are discussed in the “Numerical experiments” section. “Conclusions” are drawn in the final section.

## Related works

### Scheduling algorithms

In a manufacturing system, scheduling is regarded as an effective way to save energy and improve production efficiency by allocating limited resources to tasks (jobs). Machines are the main resources in production. The whole production process in the iron and steel manufacturing system is complex, as it involves numerous production steps with special operation requirements, so scheduling problems for different parts are often modeled and solved separately. For example, the steel-making continuous casting scheduling problem is a specific case of the hybrid flow shop scheduling problem accompanied by technological constraints of steel-making^7^, and the hot rolling scheduling problem can be modeled as a multiple traveling salesman problem based on actual production constraints^8^.

This paper considers the scheduling problem between the iron- and steel-making processes, which is actually a special case of a non-identical parallel-machine scheduling problem (PMSP) with minimizing total completion times. Li et al.^9^ reviewed that most non-identical parallel machine problems in this type are NP-hard in strong sense, and Bruno et al.^10^ showed that the problem is NP-hard for the case of even two identical machines. Therefore, the emphasis on the solution has recently shifted towards heuristic methods that can achieve a satisfactory near-optimal solution in a short time by exploring a small portion of the solution space. These solutions can be classified into three main types: dispatching rules, approximation algorithms, and meta-heuristics.

Dispatching rules include those easy rules to be implemented even by hand, e.g., SWPT rule^11^, but their weakness is also obvious: The quality of the solution can not be guaranteed. Approximation algorithms are polynomial-time algorithms that have a performance guarantee of the optimality ratio. A series of studies presented algorithms with ratio O(1) (see summary in the review^9^). Skutella^12^ gave the best known result so far, 1.2752, for the special case of two machines. In spite of the efficiency of these approximation algorithms, their optimality gaps are not negligible.

Meta heuristics are powerful methods to solve combinatorial optimization problems, which most scheduling problems can be equivalently transformed to. They are based on a similar scheme that starts from an initial solution (usually constructed by dispatching rules) and proceeds to a local optimal one through iterations. In addition, they usually contain a global search operation to avoid local optimum. One can obtain a satisfying solution in an acceptable time by designing specific meta-heuristic strategies, solution representations, and generations according to the characteristics of the problem. Thus, they have been applied in many optimization fields, such as parameter optimization in aerospace component alloys^13,14^. A variety of meta-heuristic algorithms have also been developed for different specific parallel-machine scheduling problems, such as problems with sequence-dependent setup times using VNS^15,16^, problems with resource constraints using simulated annealing algorithm^17^, and minimization of total tardiness using genetic algorithm^18^.

Variable neighborhood search method^19^ is one of the powerful meta-heuristic methods to deal with combinatorial optimization problems. By defining several neighborhood operations, VNS searches all the neighbors of the current solution to iteratively obtain a local minimum solution, and a shake (jump) operation could help it jump out of it to search for a better solution globally. When the defined neighborhood region covers the whole feasible region, an optimal solution could be obtained. Qiu et al.^20^ presented it for the production routing problem. Pan et al.^21^ developed two VNS methods to solve the distributed assembly permutation flow shop scheduling problem. Lei et al.^22^ utilized it to solve a flexible job shop scheduling problem. The reader is referred to the review^23^ for a detailed account of the literature.

### Machine scheduling under uncertainty

A common approach to deal with uncertainty in machine scheduling problems is stochastic programming (SP), which assumes that the distributions of random variables are known (like normal^24^ or exponential^25^ distribution). To enhance the robustness and hedge against the risk of unacceptable scenarios, Ranjbar et al.^26^ and Pishevar et al.^27^ applied β-robust method to the parallel machine scheduling problem, in which the processing time follows a normal distribution and the likelihood of exceeding some performance measure is minimized. However, all these methods require the exact distribution of random parameters, which is hard to obtain in practice.

Another approach to deal with the uncertainty in machine scheduling is fuzzy set theory? This theory has been applied to non-identical PMSPs with fuzzy processing times^28,29^. Fazel et al.^30^ also adopted the fuzzy approach to the SCC scheduling problem and solved it with a particle swarm optimization algorithm. Although these methods avoid using exact probability distributions of uncertain parameters, the stability of the solution gained by these models is rarely analyzed in the literature.

Robust optimization (RO) is another approach to obtaining a robust solution without exact distribution information, which assumes that the uncertain parameter belongs to a given uncertainty set and optimizes the performance under the worst-case scenario in it. This approach has been applied to many scheduling problems in the steel manufacturing process, such as the SCC scheduling problem^5^ with the processing time belonging to an interval, and collaboration scheduling with uncertain hot rolling time^31^. The RO method has also been utilized in PMSPs, including identical^32,33^ PMSP, uniform^34^ PMSP, and unrelated^35^ PMSP. However, the drawback of the RO method is that it fails to make full use of distribution information obtained from historical data.

Distributionally robust approach was first proposed for an inventory problem^36^. The DR optimization approach assumes that the true distribution belongs to a family of distributions, called the ambiguity set. Based on the type of distribution information utilized by ambiguity sets, DR methods can be roughly classified into two categories: distance-based^37–39^ and moment-based^40–42^ approaches. The distance-based approach assumes that true distribution lies in a distance from an estimated distribution, while the moment-based approach assumes that some limitations of the moments are known.

Since the moment information is easy to be obtained in practice, the moment-based DR optimization method has been applied to many scheduling fields, such as energy scheduling^43^. In the machine scheduling field, Chang et al.^44^ first introduced the DR approach to the single machine scheduling problem, in which the ambiguity set contains all the distributions with the same given first and second moments. Then, Chang et al.^45^ extend it to an identical parallel machine scheduling problem with uncertain first and second moments. This paper differs from these works in the following ways: First, the considered problem is a special non-identical parallel machine scheduling problem, where the sequence and processing time of each job in this sequence on each machine is given. Second, there is a pretreatment process for each job before it is processed by the machine (converter), and the pretreatment processing time of the job is related to the allocation results. Third, the pretreatment processing times are uncertain, which leads to uncertain parameters appearing in the constraint rather than in the objective function.

## Problem statement and formulations

**Figure 1:**
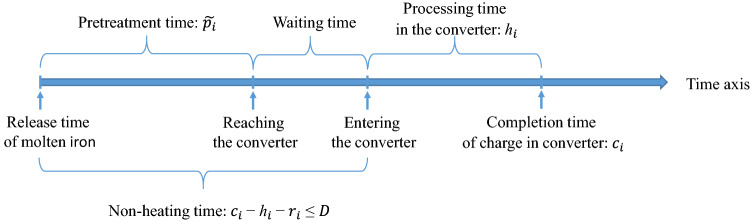
Problem illustration.

The process connecting the iron- and steel-making stage includes three main phases: iron making in the furnace, pretreatment, and the first process of steel making in the converters. The number and sequence of charges on each converter, and the processing time of each charge are obtained from a steel-making plan, which is determined according to demands before scheduling. The decisions of the iron-steel allocation problem include: (i) allocating each pot of molten iron released from the blast furnace to one of the given charges, which is a one-to-one match; and (ii) estimating the completion time of each charge in the converter. Figure 1 gives an illustration of the problem. For each pot of iron, the non-heating time, i.e., the time between iron and steel making, should not be longer than a given threshold, because the temperature of the iron will decrease, and the iron may freeze with the increase of non-heating time. The release time of each molten iron pot from the blast furnace is known, but the pretreatment time is uncertain. The only known information about the pretreatment time is its mean and variance, which are relative to the composition requirement of the charge. The optimization model of this problem aims to minimize the sum of the weighted completion times, while the production should be ensured on time with a certain probability. Relevant parameters and decision variables are listed in Table 1.

**Table 1:** Notations.

Symbol	Description
**Parameters**	
${\mathbb {J}}$	The set of molten iron pots, ${\mathbb {J}}=\{1,2,\ldots ,J\}$, where *J* is the number of pots.
${\mathbb {I}}$	The set of charges, ${\mathbb {I}}=\{1,2,\ldots ,J\}$, *J* is the total number of charges, which is the same as that of molten iron pots.
${\mathbb {M}}$	The set of converters, ${\mathbb {M}}=\{1,2,\ldots ,M\}$, where *M* is the total number of converters.
$N_{m}$	The number of charges to be processed in the *m*th converter.
$h_{mi}$	The processing time of the $i$th charge in the $m$th converter.
$w_{mi}$	The weight of the $i$th charge in the $m$th converter.
$r_j$	The release time of the $j$th pot of molten iron.
*D*	The longest allowed non-heating time between the iron- and steel-making stage.
$p_{mi}$	The pretreatment time of the $i$th charge on the $m$th converter (including transportation time).
**Decisions**	
$x_{mij}$	0–1 variable, which equals to 1 if the *j*th pot of molten iron corresponds to the $i$th charge of the *m*th converter and 0 otherwise.
$c_{mi}$	Continuous variable, the completion time of the *i*th charge of the *m*th converter.

### Nominal model

Before considering the random case, the mathematical formulation of the nominal iron-steel allocation (ISA) problem is given as follows.

1a $$\begin{aligned} \text {(ISA)}~~ \min _{\varvec{x},\varvec{c}}~&\sum _{m\in {\mathbb {M}}}\sum _{i=1}^{N_m}{w_{mi}\cdot c_{mi}} \end{aligned}$$

1b $$\begin{aligned} ~\text {s.t.}\ \&\sum _{m\in {\mathbb {M}}}\sum _{i=1}^{N_m} x_{mij}=1,~~\forall j\in {\mathbb {J}},\end{aligned}$$

1c $$\begin{aligned}\sum _{j\in {\mathbb {J}}}x_{mij}=1,~~\forall m\in {\mathbb {M}},~~\forall i=1,\ldots ,N_m,\end{aligned}$$

1d $$\begin{aligned}~c_{mi}-h_{mi}-\sum _{j\in {\mathbb {J}}}x_{mij}\cdot r_j\le D,~~\forall m\in {\mathbb {M}},~~\forall i=1,\ldots ,N_m,\end{aligned}$$

1e $$\begin{aligned}~c_{mi}\ge c_{m(i-1)}+h_{mi},~~\forall m\in {\mathbb {M}},~~\forall i=2,\ldots ,N_m,\end{aligned}$$

1f $$\begin{aligned}~c_{mi}\ge \sum _{j\in {\mathbb {J}}}x_{mij}\cdot r_j+{p}_{mi}+h_{mi},~~\forall m\in {\mathbb {M}},~~\forall i=1,\ldots ,N_m,~~~~~~~~~~~~\end{aligned}$$

1g $$\begin{aligned}~c_{mi}\ge 0,~~\forall m\in {\mathbb {M}},~~\forall i=1,\ldots ,N_m,\end{aligned}$$

1h $$\begin{aligned}~x_{mij}\in \{0,1\},~~\forall m\in {\mathbb {M}},~~\forall i=1,\ldots ,N_m,~~\forall j\in {\mathbb {J}}. \end{aligned}$$

The objective function (1a) is the total weighted completion time. Constraints (1b) and (1c) impose that a pot of molten iron can only be allocated to a charge, and a charge only corresponds to one pot. Constraint (1d) prevents waiting time over long. Constraint (1e) is the precedence constraint, i.e., one machine can only process at most one charge at any time, and only when the previous charge is finished can the next one begin. Constraint (1f) indicates that only molten iron has arrived at the converter can it begin to be processed.

### Distributionally robust optimization model

To deal with the uncertain pretreatment time and provide a reliable scheduling plan, a distributionally robust optimization model with chance constraints is considered. The uncertain pretreatment time for the *i*th charge on the *m*th converter is denoted as ${\tilde{p}}_{mi}$, and the vector form of it is $\tilde{\varvec{p}}\in {\mathbb {R}}^{J}_{+}$. The first and second moments of the probability distribution are assumed to be known, and the distribution belongs to an *ambiguity set* defined in (2).

2 $$\begin{aligned} {\mathscr {D}}^{is}=\left\{ F\ |\ {\mathbb {P}}\left( \tilde{\varvec{p}}\in {\mathbb {R}}^J_{+}\right) =1,\ {\mathbb {E}}[\tilde{\varvec{p}}]=\varvec{\mu }\ ,\ {\mathbb {V}}\text {ar}[\tilde{\varvec{p}}]=\varvec{\sigma }^2\right\} , \end{aligned}$$

where $\varvec{\mu }$ and $\varvec{\sigma }^2$ are the mean and variance of random vector $\tilde{\varvec{p}}$, and elements $\mu _{mi}$ and $\sigma ^2_{mi}$ are the mean and variance of element ${\tilde{p}}_{mi}$.

As ${\mathscr {D}}^{is}$ does not consider the correlation between elements, the marginal ambiguity set of element ${\tilde{p}}_{mi}$ is

3 $$\begin{aligned} {\mathscr {D}}^{mi}=\left\{ F^{mi}|\ {\mathbb {P}}\left( {\tilde{p}}_{mi}\in {\mathbb {R}}_{+}\right) =1,\ {\mathbb {E}}\left[ {\tilde{p}}_{mi}\right] =\mu _{mi},\ {\mathbb {V}}\text {ar}\left[ {\tilde{p}}_{mi}\right] =\sigma _{mi}^2\right\} . \end{aligned}$$

As the pretreatment time is uncertain, constraint (1f) can not be utilized anymore. Therefore it is replaced by distributionally robust chance constraints, i.e.,

4 $$\begin{aligned} \inf _{F\in {\mathscr {D}}^{mi}}~{\mathbb {P}}\left( c_{mi}\ge \sum _{j\in {\mathbb {J}}}x_{mij}\cdot r_j+{\tilde{p}}_{mi}+h_{mi}\right) \ge \alpha ,~~\forall m\in {\mathbb {M}},~~\forall i=1,\ldots ,N_m, \end{aligned}$$

which indicates that each constraint should hold with probability no less than $\alpha$ for any distribution in ${\mathscr {D}}^{is}$. Notice that the relevance effects between constraints are ignored here. Thus, when constraint (1f) is replaced by constraint (4), the resulting model is an *individual* chance-constrained model. To solve this model with off-the-shelf solvers, these stochastic constraints are transformed into deterministic ones as given in Theorem 1.

#### Theorem 1

*Given different *$\alpha$, *chance constraint* (4) *can be equivalent reformulated to the following linear constraint:*

5 $$\begin{aligned} {\left\{ \begin{array}{ll} \displaystyle \frac{\mu _{mi}}{1-\alpha }\le c_{mi}-h_{mi}-\sum _{j\in {\mathbb {J}}}x_{mij}\cdot r_j,\ &{}\text {if}\ \ 0\le \alpha \le \displaystyle \frac{\sigma _{mi}^2}{\sigma _{mi}^2+\mu _{mi}^2},\\ \mu _{mi}+\sqrt{\displaystyle \frac{\alpha }{1-\alpha }} \cdot \sigma _{mi}\le c_{mi}-h_{mi}-\displaystyle \sum \limits _{j\in {\mathbb {J}}}x_{mij}\cdot r_j,\ &{}\text {if}\ \ \displaystyle \frac{\sigma _{mi}^2}{\sigma _{mi}^2+\mu _{mi}^2}\le \alpha \le 1.\\ \end{array}\right. } \end{aligned}$$

#### Proof

Let $L_{mi}(\cdot )$ : ${\mathbb {R}}\rightarrow {\mathbb {R}}$ be a continuous loss function and defined as

6 $$\begin{aligned} L_{mi}({\tilde{p}}_{mi})={\tilde{p}}_{mi}+h_{mi}+\sum _{j\in {\mathbb {J}}}x_{mij}\cdot r_j-c_{mi},~~\forall m\in {\mathbb {M}},~~\forall i=1,\ldots ,N_m. \end{aligned}$$

According to Theorem 2.2 in the work of Zymler et al.^46^,

7 $$\begin{aligned} \inf _{F\in {\mathscr {P}}}{\mathbb {P}}\Big (L_{mi}({\tilde{p}}_{mi})\le 0\Big )\ge \alpha ~\Longleftrightarrow ~ \sup _{F\in {\mathscr {P}}}\text {CVaR}_\alpha \Big (L_{mi}({\tilde{p}}_{mi})\Big )\le 0 , \end{aligned}$$

where

8 $$\begin{aligned} \text {CVaR}_\alpha ({\tilde{z}}):={\mathbb {E}}\left[ {\tilde{z}}\ |\ {\tilde{z}}\ge \text {inf}\left\{ z:\text {Prob}\left( {\tilde{z}}>z\right) \le 1-\alpha \right\} \right] . \end{aligned}$$

Then, by Theorem 1 in Chang et al.^44^, the claim follows. $\square$

By introducing parameter $v_{mi}$ to indicate the relationship between $\alpha$ and $(\mu _{mi},\sigma ^2_{mi})$, the distributionally robust model is reformulated into the following mixed-integer programming:

9a $$\begin{aligned} \text {(DRISA)}~\min _{\varvec{x},\varvec{c}}~&\sum _{m\in {\mathbb {M}}}\sum _{i=1}^{N_m}{w_{mi}\cdot c_{mi}} \end{aligned}$$

9b $$\begin{aligned} ~\text {s.t.}\ \&\sum _{m\in {\mathbb {M}}}\sum _{i=1}^{N_m} x_{mij}=1,~~\forall j\in {\mathbb {J}}, \end{aligned}$$

9c $$\begin{aligned}\sum _{j\in {\mathbb {J}}}x_{mij}=1,~~\forall m\in {\mathbb {M}},~~\forall i=1,\ldots ,N_m,\end{aligned}$$

9d $$\begin{aligned}~c_{mi}-h_{mi}-\sum _{j\in {\mathbb {J}}}x_{mij}\cdot r_j\le D,~~\forall m\in {\mathbb {M}},~~\forall i=1,\ldots ,N_m, \end{aligned}$$

9e $$\begin{aligned}~c_{mi}\ge c_{m(i-1)}+h_{mi},~~\forall m\in {\mathbb {M}},~~\forall i=2,\ldots ,N_m,\end{aligned}$$

9f $$\begin{aligned}~c_{mi}\ge \sum _{j\in {\mathbb {J}}}x_{mij}\cdot r_j+\frac{\mu _{mi}}{1-\alpha }+h_{mi}-B(1-v_{mi}),~~\forall m\in {\mathbb {M}},~~\forall i=1,\ldots ,N_m,\end{aligned}$$

9g $$\begin{aligned}~c_{mi}\ge \sum _{j\in {\mathbb {J}}}x_{mij}\cdot r_j+\mu _{mi}+\beta \cdot \sigma _{mi}+h_{mi}-Bv_{mi},~~\forall m\in {\mathbb {M}},~~\forall i=1,\ldots ,N_m,\end{aligned}$$

9h $$\begin{aligned}~c_{mi}\ge 0,~~\forall m\in {\mathbb {M}},~~\forall i=1,\ldots ,N_m,\end{aligned}$$

9i $$\begin{aligned}~x_{mij}\in \{0,1\},~~\forall m\in {\mathbb {M}},~~\forall i=1,\ldots ,N_m,~~\forall j\in {\mathbb {J}}. \end{aligned}$$

where $\beta =\sqrt{\alpha /(1-\alpha )}$. If $\alpha \le \sigma _{mi}^2/(\sigma _{mi}^2+\mu _{mi}^2)$ then set $v_{mi}=1$, and constraint (9g) can be removed , otherwise $v_{mi}=0$ and constraint (9f) is removed. Parameter *B* is a large number such that when $v_{mi}$ takes 0 and 1, the right-hand sides of (9f) and (9g) are less than 0, respectively.

The DRISA model consists of $J^2$ binary variables and *J* continuous variables. When the problem size is small, solvers can obtain the optimal solution efficiently. However, when it comes to a large scale, solvers fail to find an optimal solution in an acceptable amount of time. To solve real-world problems efficiently, in the next section, the DRISA model will be analyzed and decomposed into a master problem and subproblem, which are solved iteratively to reach a near-optimal solution in a short time when a large-scale problem is met.

## Solution algorithm

Notice that when binary variables $\varvec{x}$ are given, only continuous variables $\varvec{c}$ are left, and the optimization model reduces to a linear program. Therefore, the model is decomposed into a master problem and a subproblem. In the master problem, a VNS method is utilized to determine the allocation of each pot to each charge (i.e., $\varvec{x}$). In the subproblem, $\varvec{x}$ is fixed and a linear program will be solved to obtain the optimal completion time (i.e., $\varvec{c}$).

### Linear program subproblem

Let $\varvec{\pi }\in {\mathbb {N}}^J_+$ be a permutation of integers $(1,\ldots ,J)$. A feasible allocation solution $\varvec{x}$ can be represented using $\varvec{\pi }$: The charges in the first converter are numbered as $1,\ldots ,N_1$, and those in the second are numbered as $N_1+1,\ldots ,N_1+N_2$, and the rest can be done in the same manner. Example 1 illustrate the correspondence between $\varvec{\pi }$ and $\varvec{x}^{\varvec{\pi }}$.

#### Example 1

For a DRISA problem with 5 pots of molten iron and two converters, the first converter consists of 3 charges, and 2 does the second. A feasible allocation solution $\varvec{A}_e$ is given as

$$\begin{aligned} (\varvec{A}_e)\ \ \ \text {Converter} \; 1{:}\;\text {Pot}\ 3-\text {Pot}\ 5-\text {Pot}\ 2\ ;\ \ \ \text {Converter}\; 2{:}\;\;\text {Pot}\ 4-\text {Pot}\ 1. \end{aligned}$$

By the mapping strategy, the corresponding $\varvec{\pi }_e$ should be $\varvec{\pi }_e=(3,5,2,4,1)^T$, and all the elements of $\varvec{x}^{\varvec{\pi }}$ should be 0 except that elements in $\big \{x_{113},x_{125},x_{132},x_{214},x_{221}\big \}$ are all 1s.

The feasible set of $\varvec{\pi }$ can therefore be be described as

10 $$\begin{aligned} \begin{aligned} \varvec{\Pi }=\Big \{\varvec{\pi }\in {\mathbb {N}}_+^J~\vert \ \pi _k\in \left\{ 1,2,\ldots ,J\right\} ,~\forall k\in {{\mathbb {I}}} ;~\left| \{\pi _k|\ \pi _k=j,\ \forall k\in {{\mathbb {I}}} \}\right| =1,~\forall j\in {{\mathbb {J}}}\Big \}. \end{aligned} \end{aligned}$$

Then, the subproblem is to solve the DRISA problem with fixed $\varvec{x}^{\varvec{\pi }}$, and the optimal value of the subproblem is denoted as Cost($\varvec{\pi }$), which can be used as the fitness function in the master problem to evaluate the quality of $\varvec{\pi }$. However, some $\varvec{\pi }\in \varvec{\Pi }$ may lead to the linear subproblem infeasible, i.e., there is no $\varvec{c}$ such that constraints (9d)–(9h) are satisfied simultaneously. Because such $\varvec{\pi }$ must not be the optimal solution of the DRISA, Cost($\varvec{\pi }$) is set as $+\infty$ for this case.

As the subproblem should be solved multiple times in the solution procedure, the computation efficiency of the subproblem is critical. Although this linear program can be solved by commercial solvers in a short time, a recurrence solution method is proposed, whose computation complexity is *O*(*J*), to further enhance its efficiency.

When the allocation vector $\varvec{\pi }$ is given, the iron release time of each charge from furnace (i.e., completion time of the iron-making process) is known. Let $r'_{mi}=\sum _{j\in {\mathbb {J}}}x_{mij}^{\pi }\cdot r_j$ be the iron release time of the $i$th charge of the *m*th converter. For each $m\in {\mathbb {M}}$ and $i=1,\ldots ,N_m$, a parameter is introduced as

11 $$\begin{aligned} a_{mi}= {\left\{ \begin{array}{ll} \displaystyle \frac{\mu _{mi}}{1-\alpha },\ &{}\text {if}\ \ 0\le \alpha \le \displaystyle \frac{\sigma _{mi}^2}{\sigma _{mi}^2+\mu _{mi}^2},\\ \mu _{mi}+\beta \cdot \sigma _{mi},\ &{}\text {if}\ \ \displaystyle \frac{\sigma _{mi}^2}{\sigma _{mi}^2+\mu _{mi}^2}\le \alpha \le 1,\\ \end{array}\right. } \end{aligned}$$

Then, constraints (9d)–(9g) can be rewritten as

12 $$\begin{aligned}~c_{mi}-h_{mi}-r'_{mi}\le D,~~~~\forall m\in {\mathbb {M}},~~\forall i=1,\ldots ,N_m,\end{aligned}$$

13 $$\begin{aligned}~c_{mi}\ge c_{m(i-1)}+h_{mi},~~~~~~\forall m\in {\mathbb {M}},~~\forall i=2,\ldots ,N_m,\end{aligned}$$

14 $$\begin{aligned}~c_{mi}\ge r'_{mi}+a_{mi}+h_{mi},~~\forall m\in {\mathbb {M}},~~\forall i=1,\ldots ,N_m. \end{aligned}$$

Notice that the objective of the subproblem is to minimized the weighted completion time. Thus, a small $c_{mi}$ is better than a big one. If there is an optimal solution of the subproblem, according to (13)–(14), the optimal solution $c^*_{mi}$ for each $m\in {\mathbb {M}}$ and $i=1,\ldots ,N_m$ can be obtained by the following recurrences:

15 $$\begin{aligned}c_{m1}^*=r'_{m1}+a_{m1}+h_{m1}, \end{aligned}$$

16 $$\begin{aligned}c_{m2}^*=\max \left\{ r'_{m2}+a_{m2}+h_{m2},~c_{m1}+h_{m2}\right\} ,\end{aligned}$$

17 $$\begin{aligned}~~\vdots \end{aligned}$$

18 $$\begin{aligned}c_{mN_m}^*=\max \left\{ r'_{mN_m}+a_{mN_m}+h_{mN_m},~c_{m(N_m-1)}+h_{mN_m}\right\} . \end{aligned}$$

If the obtained $\varvec{c}^*$ satisfies (12), it can be plugged into the objective function (9a) and then obtain the corresponding optimal value Cost ($\varvec{\pi }$) to evaluate $\varvec{\pi }$; Otherwise, the given $\varvec{\pi }$ is infeasible and Cost($\varvec{\pi }$) is set to $=+\infty$.

### Variable neighborhood search method

After decomposition, the master problem can be viewed as a combinatorial optimization problem aiming at minimizing the fitness function Cost($\varvec{\pi }$). Variable neighborhood search (VNS) method^19^ is one of the common ways to obtain a satisfactory solution efficiently for combinatorial optimization problems. To avoid the limitation of using a single neighborhood search operator, two operators are employed to search $\varvec{\pi }\in \varvec{\Pi }$, which are defined as follows.

#### Definition 1

(*Swap Operator*) Swap(*k*, *j*) refers to swapping elements at positions *k* and *j* in $\varvec{\pi }^c$. When the current solution $\varvec{\pi }$ is given, the neighborhood solution $\varvec{\pi }^{swap(k,j)}$ obtained by using Swap(*k*, *j*) is

19 $$\begin{aligned} {\pi }^{swap(k,j)}_i= {\left\{ \begin{array}{ll} \pi ^c_k,\ &{}\text {if}\ i=j,\\ \pi ^c_j,\ &{}\text {if}\ i=k,\\ \pi ^c_i,\ &{}\text {otherwise}.\\ \end{array}\right. } \end{aligned}$$

#### Definition 2

(*Insertion Operator*) Ins($k,j_1,j_2$) ($j_1\le j_2\le J$) refers to removing elements from positions $j_1$ to $j_2$ in $\varvec{\pi }$, and then inserting them into another specified position *p* where $p=min\{k,J-(j_2-j_1+1)\}$. When the current solution $\varvec{\pi }^c$ is given, let

20 $$\begin{aligned} {\pi }''_i= {\left\{ \begin{array}{ll} \pi '_i,\ &{}\text {if}\ \ i<p,\\ \pi ^c_{j_1+i-p},\ &{}\text {if}\ \ p\le i\le p+j_2-j_1,\\ \pi '_{i-(j_2-j_1+1)},\ &{}\text {if}\ \ p+j_2-j_1<i\le J\\ \end{array}\right. } \text {, where } \pi '_i= {\left\{ \begin{array}{ll} \pi ^c_i,\ &{}\text {if}\ i<j_1,\\ \pi ^c_{i+j_2-j_1+1},\ &{}\text {if}\ j_1\le i\le J-(j_2-j_1+1).\\ \end{array}\right. } \end{aligned}$$

The intermediate solution $\varvec{\pi }''$ indicates which converter will each pot belong to. Then, the pots in each converter are sorted according to the ascending order of their release time *r*. The resulting solution is the neighborhood solution $\varvec{\pi }^{ins(k,j_1,j_2)}$ obtained from the insertion operator Ins($k,j_1,j_2$).

Notice that the neighborhood size of the swap operator is $O(J^2)$ (because $k,j\in {\mathbb {J}}$), while that of the insertion operator is $O(J^3)$. The size of the insertion operator is shrunk to $O(J^2)$ via choosing one of its position parameters $j_2$ randomly, called *random insertion operator*, which is defined in Definition 3.

#### Definition 3

(*Random Insertion Operator*) Rins(*k*, *j*) refers to operator Ins$(k,j,j_2)$ where $j_2$ can be any integer in [*j*, *J*]. When the current solution $\varvec{\pi }^c$ is given, the neighborhood solution $\varvec{\pi }^{rins(k,j)}$ obtained by Rins(*k*, *j*) is an element randomly chosen from $\{\varvec{\pi }^{ins(k,j,j)},\varvec{\pi }^{ins(k,j,j+1)}, \ldots ,\varvec{\pi }^{ins(k,j,J)}\}$.

Now, a VNS algorithm is designed to solve the DRISA model based on the swap and random insertion neighborhood operators. The basic VNS algorithm starts with an initial allocation solution and searches for a local optimal solution in the neighborhood region of each operator. The current optimal solution is updated through iteration. The implementation order of these operators is denoted as *Order*, which is either $\{$Swap, Rins$\}$ or $\{$Rins, Swap$\}$, and it is determined according to the pre-experiment results. Based on this definition, the VNS algorithm is shown in Algorithm 1, where the random permutation $\varvec{c}$ plays a role in reducing the repetitiveness of the search process. 
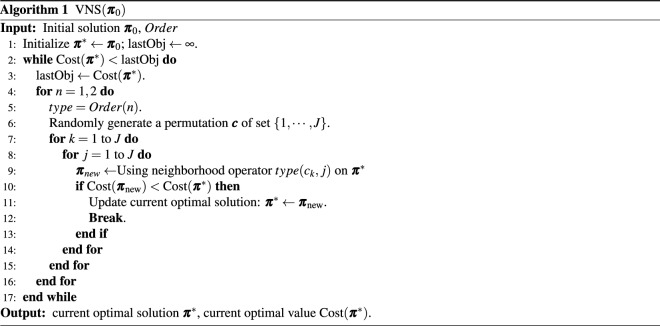


There are two initial solutions to be chosen. The pots of molten iron are arranged in ascending order to their release time from the furnaces. In the first initial solution, $1,\ldots ,N_1$ pots are assigned to the $1,\ldots ,N_1$ charges in the first converter, and $N_1,\ldots ,N_1+N_2$ pots to those in the second, and the rest is analogous. In the second initial solution, the $1,\ldots , M$ pots are assigned to the first charge of each converter, and the $M+1, \ldots ,2M$ pots to the second charge of each converter in the same turn, and the rest can be down in the same manner. Which initial solution will be used should also be determined through the results of pre-experiment.

The basic VNS algorithm shown in Algorithm 1 terminates when no better solution is found in a certain iteration, where the *iteration* here refers to a single execution from Step 2 to 17. This may lead to premature convergence to a local optimal solution and affect the algorithm’s performance. To tackle it, a jump operation is added to the basic VNS algorithm and named the jump variable neighborhood search (JVNS) algorithm. In this algorithm, when a better solution is not found at that iteration, then a neighborhood operator is employed to implement and continue to search, where the element or position to be executed can be randomly selected in jump operation. The algorithm terminates when the current solution has not been updated for a certain number of jumps. Random insertion is chosen as the jump operation because the neighborhood of it can be obtained through several swaps, which means the distance between a new solution and the current one is farther than the swap. Moreover, the randomness of random insertion makes it more likely to jump out of the local optimum. While the JVNS algorithm requires more iterations and takes longer, it has more opportunities for a better solution than the VNS algorithm.

## Numerical experiments

In this section, the pre-experiment of the VNS algorithm is implemented first to analyze the order of the neighborhood operators and the effect of the initial solution, so as to determine the settings in the following experiments. Then, production data is utilized to analyze the performance of the VNS algorithm and the stability improvement of the DRISA model. All the algorithms were coded in $\hbox {Matlab}^\circledR$ (version R2018b(9.0.5.944444) for Windows x64), and experiments were conducted on a personal computer with an $\hbox {Intel}^\circledR$ $\hbox {Core}^\text {TM}$ i7-8700 3.20GHz CPU and 16 gigabyte RAM.

Data used in experiments are all derived from a steel company in China. The number of converters is 3, and the set of the numbers of molten iron pots is {10, 20, 30, 40, 50, 100, 200}, and the corresponding numbers of charges in converters are {4,3,3}, {7,7,6}, {10,10,10}, {14,13,13}, {17,17,16}, {34,33,33}, {67,67,66}.

There is a total of twelve steel grades, each has a corresponding mean and standard deviation of the pretreatment time, and Table 2 shows this information collected from 1822 production records. In the following experiments, the steel grade of each charge is randomly selected among these 12 grades. The release time of each molten iron pot is obtained from the actual data according to the required number of pots. The average value and standard deviation of intervals between any consecutive pots are about 21.4 and 32.2, respectively. The processing time in the converter is randomly generated in [20, 30] for the diversity of instances, and the weight of each charge is randomly selected from $\{1,2,3\}$. A problem instance corresponds to a tuple ($\varvec{\mu },\varvec{\sigma },\varvec{w},\varvec{h}$).

**Table 2:** Means and standard deviation of pretreatment time.

Steel grade	1	2	3	4	5	6	7	8	9	10	11	12
$\mu$	61.43	58.66	53.35	54.01	67.31	63.91	59.29	60.40	50.00	57.83	42.40	51.80
$\sigma$	18.46	13.22	14.01	19.07	24.02	17.26	21.16	13.56	12.98	10.34	9.02	14.97

### Pre-experiment

To investigate the best settings in the VNS algorithm, pre-experiments are carried out to study the effect of different operator orders and initial solutions. $Order=\{$Swap,Rins$\}$ is numbered as Order 1 and $Order=\{$Rind,Swap$\}$ as Order 2, and the first initial solution described in the Variable neighborhood search method subsection is numbered as Solution 1 and the second as Solution 2.

In this experiment, 100 instances are randomly generated with $J=20$, $\alpha =0.90$ to test the performance. The averaged results on these instances are shown in Table 3. The results reveal that the performance of each Order-Solution combination is not much different, and Order 2 with initial solution 2 is slightly better than others in both optimality and efficiency. Hence, the following experiments are conducted with the first initial solution and operator $Order=\{$Rins, Swap$\}$. However, there is a drawback of Solution 1, i.e., Solution 1 might be infeasible, especially when in large problem, because Solution 1 tends to arrange pots of molten iron continuously released from the blast furnaces in a period of time to *one* converter, instead of distributing them separately. Therefore, in the cases when $\pi$(Solution 1)=$+\infty$, Solution 2 will be used as the initial solution.

**Table 3:** Performance of different operator orders and initial solutions ($J=20$, $\alpha =0.90$).

Order-solution	1–1	1–2	2–1	2–2
Relative gap (%)	0.52	0.49	**0.47**	0.55
Run time (in s)	0.050	0.047	**0.034**	0.035

Significant values are in bold.

### Algorithm comparison

The problem was solved by iteratively solving a master problem and subproblem. The subproblem was solved to determine the completion time for each charge on the converter. It was a linear program but could be solved by recurrence.

In this section, the performance of the VNS, JVNS algorithm, and commercial solver CPLEX will be compared in solving the DRISA problems with different problem sizes. For each problem size, 100 instances are randomly generated and used to measure the performance of different algorithms.

The maximum number of jumps in the JVNS algorithm is set to 5 for small to medium problem sizes (i.e., 10–100) and 3 for problems in large size (200) to balance the solution time and optimality, as the time that each iteration takes is growing with the problem size. If the current best solution is not updated for the maximum number of jumps in consecutive iterations (jump operation is executed at these iterations), then the algorithm terminates. The run time of the JVNS is definitely longer than that of the VNS algorithm. The run-time limit of the CPLEX in each instance is set equal to the run time used in the JVNS algorithm in the same instance, which can provide a more persuasive result. When the run-time limit is reached, the CPLEX will provide a lower bound (LB) of the optimal value, which will be used to compute the relative gap of the solution, i.e., (Cost($\pi ^*$)-LB)/LB.

The results of the comparison are shown in Table 4. In this table, V, J, and C denote VNS, JVNS, and CPLEX. V<C means the proportion of instances in which the gap of the VNS is less than the CPLEX. J<C and J<V are analogous. Only for 71% of instances with J=200 could the CPLEX work out a feasible solution in a limited time. Results excluding infeasible instances for CPLEX are also shown but in parentheses ($\cdot$) for comparison. The results show that the JVNS algorithm performs slightly better than the CPLEX when the problem size is medium (J=50,100), and the gap of VNS is close to the CPLEX while the VNS takes a shorter solution time. When it comes to a larger size, JVNS performs significantly better than CPLEX, and the CPLEX could not even find a feasible solution within a limited time for some cases. Moreover, in general, with the growth of the problem size, the advantages of JVNS over CPLEX and the improvement of the JVNS over VNS are more prominent. Considering that as long as there is sufficient time, the CPLEX can obtain solutions of arbitrary precision, one can choose the appropriate algorithm according to the requirements of time and precision in practice.

**Table 4:** Comparison between VNS, JVNS, and CPLEX.

*J*	Gap (%)			Proportion (%)			Time	
	VNS	JVNS	CPLEX	V<C	J<C	J<V	VNS	JVNS
10	0.53	**0.46**	0.54	48	48	4	0.01	0.02
20	1.99	**1.88**	2.08	57	58	14	0.03	0.13
30	2.39	**2.33**	2.35	52	55	19	0.10	0.36
40	2.91	**2.84**	2.88	43	47	26	0.24	0.84
50	2.65	**2.61**	2.98	59	61	25	0.50	1.69
100	2.26	**2.19**	2.49	64	70	59	8.54	37.02
200	3.10 (3.08)	**2.22 (1.95)**	–(2.40)	43(20)	87 (82)	62 (80)	19.08	164.18

Significant values are in bold.

### Robustness of the DRISA model

To test the robustness of the DRISA model, the performances of the schedules under different distributions are compared. 20 instances and randomly generated and the instance set is denoted as ${\mathbb {T}}$. Then, for each distribution and for each instance, 100 test samples $\tilde{\varvec{p}}$ are generated for each instance ($\varvec{\mu }$, $\varvec{\sigma }$). Performances on these test samples are observed for different settings of $\alpha$ under different distributions. When $\alpha$ is set to 0, the DRISA model is reduced to a nominal ISA model (1) with $\varvec{p}=\varvec{\mu }$. Denote $Obj^0$ as the optimal value of the nominal model.

When an instance *t* and the probability parameter $\alpha$ are given, by solving the DRISA model, a planned solution ($\varvec{x},\varvec{c}$) is obtained. Since the $\varvec{c}$ may not satisfy constraints (1d)–(1f) when the random variable $\tilde{\varvec{p}}$ is revealed, the completion time would be adjusted to an actual one $\varvec{c}^a$ for each test sample $\tilde{\varvec{p}}$, where $\varvec{c}^a$ can be obtained through the following recurrences:

21 $$\begin{aligned}c_{m1}^a=\max \left\{ c_{m1},r'_{m1}+{\tilde{p}}_{m1}+h_{m1}\right\} , \end{aligned}$$

22 $$\begin{aligned}c_{m2}^a=\max \left\{ c_{m2},r'_{m2}+{\tilde{p}}_{m2}+h_{m2},~c_{m1}+h_{m2}\right\} ,\end{aligned}$$

23 $$\begin{aligned}~~\vdots \end{aligned}$$

24 $$\begin{aligned}c_{mN_m}^*=\max \left\{ c_{mN_m},r'_{mN_m}+{\tilde{p}}_{mN_m}+h_{mN_m},~c_{m(N_m-1)}+h_{mN_m}\right\} . \end{aligned}$$

Then, the actual objective value of this sample is $\sum _{m\in {\mathbb {M}}}\sum _{i=1}^{N_m}{w_{mi}\cdot c^a_{mi}}$. $Obj^{na}$ and $Obj^{ra}$ denote the value of an actual plan adjusted from a robust plan (with a given $\alpha$) and a nominal one (with $\alpha =0$), respectively.

Four criteria are adopted to investigate the impact of the robustness factor $\alpha$: the number of charges (NC) whose completion times need to be adjusted, the total delay time (DT), the robust price (RP), and the robust revenue (RR). Let ${\mathbb {S}}_t$ be the sample set of instance *t* and generated from a certain distribution. These criteria are calculated as follows:

25 $$\begin{aligned}NC := \text {AVE}_{t\in {\mathbb {T}},s\in {\mathbb {S}}_t}(\sum _{m\in {\mathbb {M}}}\sum _{i=1}^{N_m}1_{\{c_{mi}\ne c^a_{mi}\}}),\end{aligned}$$

26 $$\begin{aligned}DT := \text {AVE}_{t\in {\mathbb {T}},s\in {\mathbb {S}}_t}\big (\sum _{m\in {\mathbb {M}}}\sum _{i=1}^{N_m} (c^a_{mi}-c_{mi})\big ),\end{aligned}$$

27 $$\begin{aligned}RP := \frac{\text {AVE}_{t\in {\mathbb {T}},s\in {\mathbb {S}}_t}(Obj^{ra}-Obj^{na})}{\text {AVE}_{t\in {\mathbb {T}},s\in {\mathbb {S}}_t}(Obj^{na)}},\end{aligned}$$

28 $$\begin{aligned}RR := \frac{\text {AVE}_{t\in {\mathbb {T}}}(\text {STD}_{s\in {\mathbb {S}}_t}(Obj^{na})-\text {STD}_{s\in {\mathbb {S}}_t}(Obj^{ra}))}{\text {AVE}_{t\in {\mathbb {T}}}(\text {STD}_{s\in {\mathbb {S}}_t}(Obj^{na)})}, \end{aligned}$$

where AVE$(\cdot )$ and STD$(\cdot )$ are the mean value and standard deviation over instances or samples described in the subscripts.

**Figure 2:**
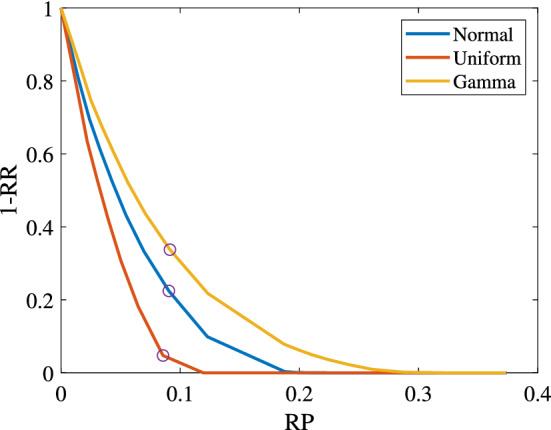
Relation of RP and RR.

Table 5 depicts the performance of the DRISA model under different $\alpha$s with $J=20$. In the nominal ISA model ($\alpha =0$), over $\frac{1}{4}$ of the charges need to be adjusted, and the average total deviation of the plan is over 70 min. Such frequent adjustments will bring great instability to the production process, and will also affect the normal progress of subsequent continuous casting and hot rolling processes. As $\alpha$ increases, both the number of charges and length of time that have to be adjusted in the implementation decrease, and the decreasing standard deviations (increasing RR) also indicate the improving robustness of the plan. Since the robustness is obtained by sacrificing a certain production efficiency (i.e., by prolonging the completion time in the plan), a trade off between the robustness and efficiency can be made by choosing a proper $\alpha$ in practice. For example, in the case $J=20$ shown in Table 5, $\alpha =0.7$ seems to be a good choice with sacrificing less than 10% RP to cut off over half of the standard deviation. Figure 2 plots part of the information in Table 5, and circles the points with $\alpha =0.7$.

**Table 5:** Performance under different $\alpha$s ($J=20$).

$\alpha$	Normal				Uniform				Gamma			
	RP (%)	RR (%)	NC	DT	RP (%)	RR (%)	NC	DT	RP (%)	RR (%)	NC	DT
0.00	0.00	0.00	5.52	73.14	0.00	0.00	5.68	78.72	0.00	0.00	5.18	74.23
0.10	1.45	19.31	4.17	46.98	1.37	22.55	4.59	51.33	1.57	15.59	3.93	50.40
0.20	2.39	30.48	3.50	36.16	2.20	36.59	4.00	38.72	2.52	25.40	3.29	39.87
0.30	3.28	38.83	2.93	28.09	3.03	46.98	3.48	29.43	3.44	32.55	2.79	32.33
0.40	4.28	47.49	2.38	21.21	3.94	57.71	2.94	20.88	4.44	39.76	2.34	25.70
0.50	5.45	56.78	1.82	15.08	5.03	69.32	2.31	13.04	5.60	47.78	1.86	19.47
0.60	6.95	66.67	1.28	9.50	6.47	81.79	1.52	6.14	7.08	56.40	1.36	13.71
0.70	9.05	77.55	0.72	4.75	8.56	95.25	0.59	0.98	9.14	66.24	0.87	8.38
0.80	12.29	90.12	0.26	1.33	11.94	100.00	0.00	0.00	12.32	78.24	0.41	3.73
0.90	18.77	99.67	0.01	0.02	18.49	100.00	0.00	0.00	18.71	92.13	0.06	0.58
0.91	19.83	99.92	0.00	0.01	19.56	100.00	0.00	0.00	19.77	93.52	0.05	0.42
0.92	21.04	99.97	0.00	0.00	20.77	100.00	0.00	0.00	20.97	94.95	0.03	0.28
0.93	22.45	99.99	0.00	0.00	22.19	100.00	0.00	0.00	22.38	96.33	0.02	0.18
0.94	24.14	100.00	0.00	0.00	23.89	100.00	0.00	0.00	24.08	97.70	0.01	0.10
0.95	26.21	100.00	0.00	0.00	25.96	100.00	0.00	0.00	26.14	99.06	0.01	0.04
0.96	28.82	100.00	0.00	0.00	28.58	100.00	0.00	0.00	28.75	99.82	0.00	0.01
0.97	32.28	100.00	0.00	0.00	32.06	100.00	0.00	0.00	32.22	100.00	0.00	0.00
0.98	37.40	100.00	0.00	0.00	37.19	100.00	0.00	0.00	37.34	100.00	0.00	0.00

## Conclusions

This paper studied the iron-steel allocation problem where the pretreatment time was uncertain with known mean and variance, and a distributionally robust model was constructed with chance constraints. The true distribution of the pretreatment time vector was assumed to belong to an ambiguity set, which consisted of all the distributions with the given mean and variance. The goal of this model was to minimize the weighted completion time while the constraints with the uncertain parameters were guaranteed to hold with a required probability even under the worst-case distribution in the ambiguity set. This DR model was equivalently reformulated into a MIP model, such that it could be solved by any off-the-shelf solvers. Furthermore, the MIP model was decomposed into an integer programming master problem and a linear programming subproblem. A meta-heuristic method, VNS, for combinatorial optimization problems was utilized to speed up the solution in large-scale instances. A modified version, JVNS, with jump operation was also introduced to avoid local minimum.

Experiments based on the data from a steel company were conducted to confirm the claims. For different problem sizes, the average gaps of the JVNS were always lower than that of the CPLEX within the same solution time, and the advantage of JVNS grew prominent with the increase in problem size, which demonstrated the efficiency of JVNS in large-scale problems. Since the VNS could obtain a feasible solution in a short time and the CPLEX could obtain an exact solution as long as the solution time is sufficiently large, one could choose a proper algorithm among them based on the actual situation and requirements (e.g., time, quality, problem size). Computational results about the robustness showed that with the increase of the guaranteed probability $\alpha$, the delayed charges were fewer and the total delay time was less at a cost of more total weighted completion time. Therefore, the conclusion could be drawn that the proposed DR molten iron-steel allocation model could be used to derive a stable plan for the connecting process between iron and steel making stages, such that the subsequent processes can proceed as expected.

## Data Availability

Most of the samples generated in this article are randomly generated according to the statistics from the real data, except for the release times from the blast furnaces, but the average value and standard deviation of the release times are also given in the article. Since the statistics and ways to generate samples have been presented in the article, the authors believe that conclusions drawn by the simulations could be reproduced. The raw data of this study are available from Xinyu Iron & Steel Co., Ltd., but restrictions apply to the availability of these data, which were used under license for the current study, and so are not publicly available. Data are however available from the authors upon reasonable request and with permission of Xinyu Iron & Steel Co., Ltd.
